# Application of medical gases in prevention and treatment of electromagnetic radiation injury

**DOI:** 10.4103/mgr.MEDGASRES-D-26-00051

**Published:** 2026-09-12

**Authors:** Yun Xing, Fengxu Chen, Gonggeng Liu, Shijun Bao, Xujian Zhang, Ziyi Guo, Zhaoyong Shou, Jiaming Guo

**Affiliations:** 1 Department of Radiation Medicine, College of Naval Medicine, Naval Medical University, Shanghai, China; 2 Faculty of Health Service, Naval Medical University, Shanghai, China

**Keywords:** apoptosis, bystander effect, clinical translation, delivery strategy, electromagnetic radiation, gasotransmitter, inflammation, medical gas, oxidative stress, radioprotection

## Abstract

The widespread proliferation of electromagnetic technologies in communications, healthcare, and daily life poses increasing risks of radiation-induced tissue injury. Mitigating these deleterious effects remains a formidable challenge. Recently, medical gases, including molecular hydrogen (H_2_), hydrogen sulfide (H_2_S), carbon monoxide (CO), nitric oxide (NO), and hyperbaric oxygen, have emerged as a promising therapeutic modality with radioprotective potential. These gases exert their effects through integrated, anti-inflammatory, anti-apoptotic, and mitochondrial-protective mechanisms. Specifically, the endogenous gasotransmitters (CO, NO, and H_2_S) modulate precise signaling networks, while the exogenous interventions (H_2_, and hyperbaric oxygen) function primarily through direct radical scavenging or the induction of hormetic stress responses. This review evaluates the research progress of these five medical gases in preventing and treating injuries across the electromagnetic spectrum. H_2_ possesses the broadest evidence base, particularly in radiofrequency and ultraviolet protection. H_2_S and CO demonstrate potent efficacy in addressing ultraviolet-induced skin and retinal damage. NO plays a dual role as both a cytoprotectant and a mediator of bystander effect propagation. Hyperbaric oxygen enhances systemic resilience through preconditioning. Delivery strategies have evolved from conventional inhalation and oral administration toward smart nanocarriers and stimuli-responsive donor systems. However, these platforms still face challenges in spatiotemporal control and long-term safety. Furthermore, broader limitations, including an uneven research distribution across electromagnetic wavebands, a scarcity of clinical trials, and the lack of non-invasive methods for real-time gas concentration monitoring, are comprehensively discussed.

## Introduction

The therapeutic landscape of gaseous signaling molecules, notably hydrogen (H_2_), hydrogen sulfide (H_2_S), carbon monoxide (CO), and nitric oxide (NO), supplemented by hyperbaric oxygen (HBO) therapy, has broadened significantly, leveraging their superior tissue penetrability and biocompatibility.^1^ This class of medical gases is characterized by its capacity to modulate critical cellular signaling pathways involved in oxidative stress, inflammation, and cell survival. Among them, CO and NO are well-established endogenous gasotransmitters, whose actions are highly concentration-and context-dependent. In contrast, H_2_ and HBO primarily function as exogenous interventions. This distinction in origin underpins differences in their delivery paradigms and application strategies.

The therapeutic exploration of these gases has evolved over decades. Research on medical gases back to the 20^th^ century, and in the 21^st^ century has focused on preventing radiation damage through gas therapy (**Figure 1**). Pharmacologically, these gas molecules have been confirmed to exert selective antioxidant, antiinflammatory, and antiapoptotic effects, modulating pathways that are also central to injury mechanisms triggered by a pervasive modern challenge - electromagnetic radiation.

**Figure 1:**
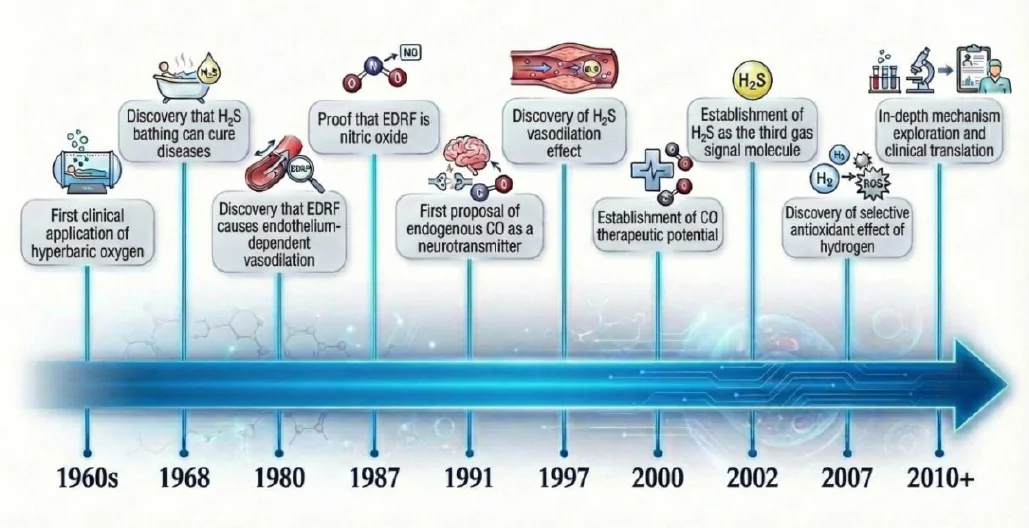
The key milestones in medical gas research. This timeline shows key discoveries and advancements from the 1960s to the present. Created with Adobe Illustrator 2025. CO: carbon monoxide; EDRF: endothelium-derived relaxing factor; H_2_: hydrogen; H_2_S: hydrogen sulfide; NO: nitric oxide.

In contemporary society, electromagnetic radiation is pervasive in various domains of daily life, including communications and healthcare. While its widespread application offers significant convenience, it leads to an escalating risk of human exposure.^2^ With increasing exposure, electromagnetic radiation has been shown to induce damage to multiple organ systems, including the nervous system, reproductive system, and skin. Current mainstream protective measures, chiefly physical shielding, are constrained by spatial and temporal constraints. In addition, effective pharmacological strategies remain largely exploratory, resulting in a clinical shortage of safe and reliable radioprotective agents.

To address this gap, the present review adopts a gas-centric, origin-based framework to propose targeted interventions for electromagnetic radiation injury. We first delineate the pathophysiological impact of electromagnetic radiation, followed by a critical evaluation of the therapeutic effects and underlying mechanisms of H_2_, H_2_S, CO, NO, and HBO. By analyzing these mechanisms through the lens of their endogenous versus exogenous nature, we provide a framework for future clinical translation.

## Literature Search

A systematic literature search was performed using the PubMed database. The formal search focused on articles published from January 2000 to March 2026. The search employed various combinations of the following terms: medical gas, hydrogen, H_2_, hydrogen sulfide, H_2_S, carbon monoxide, CO, nitric oxide, NO, hyperbaric oxygen, HBO, electromagnetic radiation, radiofrequency, radio waves, infrared, visible light, blue light, ultraviolet, UV, radioprotection, radiation injury, oxidative stress, and bystander effect. Additional terms included gasotransmitter, delivery system, nanocarrier, and clinical translation were conducted to capture relevant studies on mechanisms and applications. Titles and abstracts were screened for relevance to the topic. Articles were included if they met the following criteria: published in English, focused on the protective or therapeutic effects of medical gases against electromagnetic radiation injury or closely related oxidative/inflammatory damage, and contained original experimental data, clinical findings, or comprehensive reviews with mechanistic insights. Articles were excluded if they focused exclusively on ionizing radiation without mention of electromagnetic radiation, lacked mechanistic or protective outcome data, or were case reports, editorials, or conference abstracts without full text available. After initial screening, the full texts of potentially relevant articles were reviewed to confirm eligibility. In addition, several foundational studies published before 2000, including pioneering work from 1975, were identified through manual screening of reference lists and included to provide essential historical context. (**Figure 2**).

**Figure 2:**
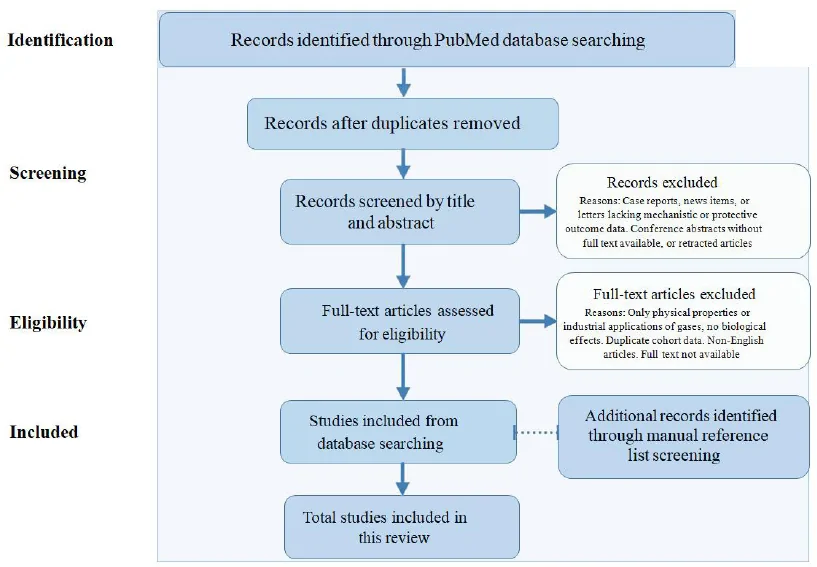
Flow diagram of the literature search and selection process. A systematic search of PubMed was performed for articles published between January 2000 and March 2026. Created with Adobe Illustrator 2025.

## Injury and Current Protective Gaps of Electromagnetic Radiation

The ubiquity of electromagnetic radiation in modern technological environments has made increasing human exposure and associated health risks inevitable.^3^ Despite the physical disparities between categories, namely radio waves (3 Hz–300 GHz), infrared radiation (300 GHz–400 THz), visible light (400–750 THz), and ultraviolet (UV; 750 THz–30 PHz), the resulting biological injuries converge on common pathways (**Figure 3**). The damage primarily stems from oxidative stress, triggered directly or indirectly by each radiation type, which leads to reactive oxygen species (ROS) overproduction, mitochondrial dysfunction, activation of inflammatory cascades (e.g., nuclear factor κB (NFκB), nucleotide-binding domain, leucine-rich repeat, and pyrin domain containing 3 (NLRP3)), and ultimately cellular demise via apoptosis, pyroptosis, or ferroptosis.^4^,^5^,^6^,^7^,^8^,^9^,^10^ Intriguingly, the bystander effect, wherein signals from irradiated cells affect neighboring non-irradiated cells, was historically considered a phenomenon exclusive to ionizing radiation. However, recent evidence suggests it also occurs following electromagnetic radiation exposure, further complicating the injury landscape and indicating the systemic nature of the damage.^11^,^12^,^13^

**Figure 3:**
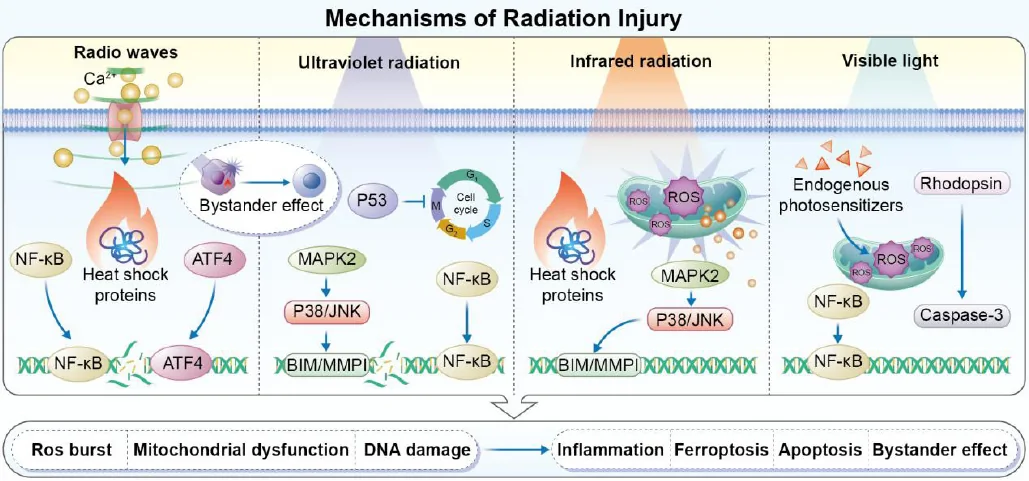
Key molecular pathways of electromagnetic radiation-induced cellular injury. Different radiation types activate specific upstream signals, which converge on core events, such as ROS burst, mitochondrial dysfunction, and activation of pathways. These processes ultimately drive downstream pathological outcomes, including inflammation, DNA damage, ferroptosis, apoptosis, and the bystander effect, illustrating the multi-level nature of radiation injury. Created with Adobe Illustrator 2025. ATF4: activating transcription factor 4; BIM: Bcl2-interacting mediator of cell death; Ca^2+^: calcium ion; JNK: c-Jun N-terminal kinase; MAPK2: mitogen-activated protein kinase 2; MMPI: matrix metalloproteinase inhibitor; NF-κB: nuclear factor κB; ROS: reactive oxygen species.

Existing medical countermeasures against these injuries remain limited and predominantly palliative (**Table 1**). While radio waves exposure is often addressed using antioxidants like melatonin or plant-derived compounds, their efficacy remains variable, and optimal pharmacological profiles are unclear.^14^,^15^,^16^,^17^ Protection against infrared radiation is particularly underdeveloped, often limited to the adjunctive use of antioxidants in topical formulations without dedicated agents.^18^ For visible light, especially high-energy blue light, interventions are sparse and focus on local antioxidant application, with long-term safety and efficacy data lacking.^19^ The most advanced measures exist for UV protection, primarily involving broad-spectrum sunscreens combining physical blockers and organic filters. However, concerns persist regarding the systemic absorption, photostability, and long-term safety of some filters, and user compliance is variable.^20^ Consequently, there is a pressing need for a new generation of protective agents that can simultaneously and precisely modulate the interconnected oxidative, inflammatory, and cell death signaling pathways implicated across the electromagnetic spectrum.

**Table 1:** Current measures of electromagnetic radiation injury

Radiation	Current mainstream countermeasure	Limitation
Radio waves	Antioxidant supplementation, e.g., melatonin, botanical extracts	1. Lack of precise molecular targets 2. Unclear optimal dosage and timing
Infrared radiation	Adjuvant use of antioxidants in topical formulations	The universal protection guidelines are impractical
Visible light	Topical application of antioxidants; Use of radical scavengers	Limited long-term safety and tolerability data
Ultraviolet radiation	Broad-spectrum sunscreens	Debated systemic absorption and long-term safety

## Protective Mechanism and Application of Hydrogen

### Protective effects across electromagnetic radiation injury models

#### Protection against radio wave radiation injury

The radioprotective efficacy of H_2_ is well-documented in models of radio waves radiation injury, H_2_ exerts synergistic protective effects on multiple sensitive systems by preserving mitochondrial integrity and alleviating oxidative stress. Pretreatment with H_2_-rich water has been shown to mitigate anxiety-like behaviors in rats exposed to radio waves and structural damage to neurons in the hippocampus and amygdala.^21^ Concurrent with this, H_2_ influences the expression of genes related to signaling pathways. For example, it upregulates glutathione S-transferase theta pseudogene 2 (Gstp2), enhancing the glutathione metabolic pathway to combat oxidative stress, and modulates multiple genes within the neuroactive ligand-receptor interaction and cyclic adenosine monophosphate signaling cascades, collectively preserving neuronal function from both metabolic and genetic perspectives. This protective signature extends to the reproductive system, where H_2_ intervention maintains spermatogonial viability and suppresses apoptosis in the face of radiation-induced germ cell injury. This effect is tied to the ability of H_2_ to scavenge ROS, restore mitochondrial function, and specifically regulate genes involved in mitochondrial antioxidant activity, further confirming its core anti-apoptotic capacity.^22^ In terms of respiratory system defense, H_2_ demonstrates protective efficacy against both acute and chronic phases of radiation-induced lung injury. It directly neutralizes the burst of hydroxyl radicals during the initial radiation exposure, thereby exerting anti-inflammatory and anti-apoptotic cascades.^23^

#### Protection against visible light injury

In visible light-induced damage, the protective role of H_2_ is primarily mediated through the silent information regulator sirtuin 1 (Sirt1) signaling pathway. By upregulating Sirt1 expression, H_2_ modulates the Bcl2/Bax protein balance, inhibits caspase3 activity, and enhances superoxide dismutase activity, thereby alleviating apoptosis and oxidative stress in photoreceptor cells.^24^ Parallel to these anti-apoptotic effects, H_2_-rich saline has been shown to expedite the functional recovery of melanopsin-mediated signaling following intense blue light exposure.^25^ While existing research on the protective or therapeutic effects of molecular H_2_ against visible light-induced damage remains limited, its demonstrated dual capacity to inhibit injury and promote functional recovery indicates its considerable potential as a gaseous protective agent.

#### Protection against ultraviolet radiation injury

H_2_ enhances cellular antioxidant defense by triggering the phosphatidylinositol 3-kinase (PI3K)/protein kinase B (Akt) signaling pathway, thereby promoting nuclear factor erythroid 2-related factor 2 (Nrf2) nuclear translocation and upregulates downstream target genes, such as heme oxygenase1.^26^ For UVA-induced skin photoaging, longterm intermittent exposure to a lowconcentration H_2_ environment effectively suppresses epidermal hyperplasia and melanin deposition while reducing levels of DNA oxidative damage markers, an effect attributable to both direct antioxidant activity and potential indirect signaling modulation.^27^,^28^ Beyond dermal protection, the radioprotective scope of H_2_ extends to UVB-induced retinal damage, a process largely driven by oxidative stress.^29^ H_2_ exerts its protective effects by virtue of its ability to effectively scavenge ROS and restore the cellular redox homeostasis.^30^ Synthetically, H_2_ primarily orchestrates enhanced endogenous antioxidant defenses and directly mitigates oxidative insult.

### Core molecular protective mechanisms

The origins of H_2_ medicine can be traced back to 1975, when high-pressure H_2_ was first reported to prevent and treat UV-induced squamous cell carcinoma in mice.^31^ In 2007, H_2_ was identified as an antioxidant capable of selectively scavenging ROS and reactive nitrogen species.^32^ In 2014, Wang^33^ proposed that, according to the criteria for gaseous messengers, H_2_S, NO, and CO qualify as gasotransmitters, whereas H_2_ could not be classified as such due to the absence of a mammalian cellular biosynthesis pathway, even though it can be produced by H_2_-generating gut bacteria. As an exogenous protective agent, H_2_ does not engage in classical receptor-mediated signaling cascades in the same way as endogenous gasotransmitters. Its benefits arise from its direct physicochemical interactions and indirect modulation of cellular defense pathways. Functioning as a therapeutic gas, H_2_ exerts multifaceted regulatory effects on signaling pathways.^34^ For instance, H_2_ reduces the generation of oxidative products by inhibiting nicotinamide adenine dinucleotide phosphate (NADPH) oxidase activity, which is achieved through attenuating the phosphorylation of Lyn (Lck/yes-related novel tyrosine kinase) protein and its downstream signal transduction.^35^ The advantage of H_2_ in mitigating oxidative stress lies in its unique antioxidant properties. Unlike conventional antioxidants, H_2_ selectively neutralizes highly reactive oxidants such as the hydroxyl radical (·OH) and peroxynitrite (ONOO^−^), thereby directly alleviating oxidative damage and maintaining redox homeostasis. A deeper mechanism involves the activation and regulation of endogenous cellular defense systems. H_2_ can modify key cysteine residues, stabilizing the Nrf2 and promoting its nuclear translocation. This, in turn, facilitates its binding to the antioxidant response element (ARE), leading to the systemic upregulation of numerous antioxidant enzymes and phase II detoxifying enzymes, thereby enhancing cellular adaptive capacity to stress.^36^ A recent study indicates that H_2_ may promote Nrf2 expression and potentiate the glutathione peroxidase 4 pathway, a primary surveillance mechanism against ferroptosis, suggesting its potential therapeutic relevance against radiation-induced ferroptosis.^37^ Beyond this pathway, H_2_ also modulates several other key signaling cascades, including NF-κB, mitogen-activated protein kinase (MAPK), and PI3K/Akt, thereby suppressing the release of pro-inflammatory cytokines and inhibiting the caspase cascade to exert broad anti-inflammatory and anti-apoptotic effects.^38^,^39^,^40^ Further evidence shows that H_2_ influences the crosstalk between autophagy and apoptosis, and improves cellular metabolic adaptability by suppressing abnormal accumulation of hypoxia-inducible factor-1α (HIF-1α) and promoting the restoration of mitochondrial function.^41^ Collectively, through both direct scavenging and indirect regulatory actions, H_2_ provides not only immediate antioxidant defense but also long-term adaptive protection.

### Delivery strategies and application challenges

Strategies aim to overcome its low solubility and achieve therapeutic concentrations through various routes, with safety primarily governed by physical limits rather than intrinsic receptor-mediated toxicity. Conventional administration routes comprise inhalation, as well as the systemic intake of H_2_-rich water or saline, and topical applications such as H_2_-rich baths and H_2_-infused eye drops. Among these, inhalation offers advantages for respiratory system protection but carries an explosion risk; oral administration of H_2_-rich water is limited by its low solubility and variable intake, which can constrain therapeutic efficacy. Technological refinements have pivoted toward optimizing both safety and delivery efficiency.^42^,^43^ Nanotechnology offers a versatile platform to surmount the challenges of H_2_’s low solubility and the lack of targeted delivery in practical applications. By employing nanomaterials that respond to external stimuli, it becomes possible to control H_2_ generation under light, ultrasound, magnetic fields, or specific microenvironmental conditions, thereby overcoming the limitations of traditional delivery methods.^44^ Nevertheless, the clinical translation of H_2_ therapy still faces multiple challenges, including precise dose regulation of delivery systems and insufficient long-term safety data.

## Protective Mechanism and Application of Hydrogen Sulfide

### Protective effects across electromagnetic radiation injury models

#### Protection against infrared radiation injury

Preliminary studies indicate that H_2_S can mitigate infrared radiation-induced damage. Regarding protection against infrared radiation, studies suggest that H_2_S can exert beneficial effects by intervening in post-irradiation cellular processes. In models where 835 nm infrared laser irradiation inhibited growth and induced cell death in human mesenchymal stem cells and melanoma cells, intervention with the donor sodium hydrosulfide (NaHS) demonstrated clear protective efficacy. This effect is likely associated with the ability of H_2_S to modulate radiation-induced signaling disruption and metabolic dysregulation.^45^ However, research on H_2_S-mediated protection against other infrared radiation effects and in-depth mechanistic exploration remains scarce, necessitating further studies to provide a solid theoretical foundation. Given that H_2_S participates in the regulation of endoplasmic reticulum stress signaling pathways through protein persulfidation to promote cellular homeostasis,^46^ it is plausible to speculate that it holds potential in mitigating protein misfolding and metabolic disturbances caused by the thermal effects of infrared radiation. Consequently, elucidating these proteostatic mechanisms remains a critical prerequisite for future experimental validation.

#### Protection against ultraviolet radiation injury

H_2_S has been demonstrated to attenuate UVA-induced cytotoxicity in normal human dermal fibroblasts, help maintain cellular bioenergetics, and promote collagen expression. The underlying molecular mechanisms align with the core pathways described earlier, involving enhanced nuclear translocation of the transcription factor Nrf2 and a systemic boost in cellular antioxidant defenses.^47^ What’s more, animal models further demonstrate that topical application of mitochondrially-targeted H_2_S donors can significantly prevent UVA-induced collagen loss and the rise of oxidative damage markers in mice, concurrently activating the Nrf2 signaling pathway.^48^ The molecular basis of these protective effects is rooted in the unique property of H_2_S to post-translationally modify target proteins through persulfidation. This modification can alter protein function, stability, and localization. After UV radiation, persulfidation of Keap1, the endogenous inhibitor of Nrf2, is a key event that facilitates Nrf2 release, nuclear translocation, and the subsequent transcriptional activation of a battery of cytoprotective genes. Beyond antioxidant induction, H_2_S-mediated persulfidation may also directly regulate enzymes involved in mitochondrial bioenergetics and collagen biosynthesis, providing a mechanistic link to its observed effects on cellular metabolism and extracellular matrix preservation.

While current evidence remains encouraging, it is largely derived from proof-of-concept studies utilizing exogenous H_2_S donors. Critical questions regarding the role of endogenous H_2_S metabolism in the physiological response to UV radiation remain open. Furthermore, the optimal therapeutic window, balancing cytoprotective signaling against potential toxicity at high concentrations, and the long-term safety profile of sustained H_2_S modulation for photoaging prevention require systematic investigation.

Collectively, despite its nascent stage, research to date indicates the potential of H_2_S in protecting against electromagnetic radiationinduced injury. Its protective effects have been experimentally confirmed in both the infrared and UV spectral regions, with mechanisms closely centered on mitigating oxidative stress and suppressing inflammatory responses.

### Core molecular protective mechanisms

H_2_S, as an important endogenous gaseous signaling molecule, plays a central role in regulating antioxidant defense, suppressing inflammatory responses, and maintaining cellular metabolic homeostasis through its unique posttranslational modification, protein persulfidation. This modification precisely modulates the stability of target proteins, thereby influencing downstream signaling networks.^49^ Based on this mechanism, H_2_S enhances antioxidant defense by directly persulfidating the Kelch-like Ech-associated protein 1 (Keap1) protein, which disrupts the stability of the Keap1cullin 3 ubiquitin ligase complex and activates the transcription factor Nrf2. Activated Nrf2 translocates into the nucleus and drives the expression of antioxidant enzyme genes, including heme oxygenase1 and glutathione synthetase, thereby systematically strengthening the cell’s capacity to maintain redox homeostasis.^50^,^51^ Similar to H_2_, H_2_S also exerts antiapoptotic effects by activating prosurvival signaling pathways such as PI3K/Akt, and cooperatively suppresses inflammation by inhibiting inflammatory pathways including NFκB.^51^ Moreover, H_2_S exhibits complex regulatory functions in cellular metabolism; it can bidirectionally modulate autophagy by modifying and regulating the activity of kinases such as adenosine monophosphate-activated protein kinase. At the mitochondrial level, low concentrations of H_2_S serve as an electron donor, regulating cytochrome c oxidase activity and influencing energy metabolism.^52^ Notably, H_2_S can also persulfidate key metabolic enzymes, affecting their function and thereby participating in cellular metabolic reprogramming.^53^ Together, these pathways constitute a sophisticated signaling network of H_2_S. The enhanced functions of antioxidation, inflammation suppression, and metabolic homeostasis maintenance provided by this network precisely target the core injury processes induced by electromagnetic radiation, offering a thorough mechanistic explanation for the cytoprotective role of H_2_S in the prevention and treatment of electromagnetic radiationinduced damage.

### Delivery strategies and application challenges

The core challenge of H_2_S translation is to achieve local, transient, and dose-controlled H_2_S delivery that replicates its physiological action while avoiding the cytotoxicity associated with sustained high concentrations. Currently, approaches primarily rely on the direct use of sulfides such as NaHS or simple organic donors, which, despite their straightforward synthesis and ability to achieve rapid H_2_S release,^54^ suffer from uncontrolled release and poor targeting.^55^ Recent research has increasingly focused on smart delivery systems, which include both endogenous and exogenous stimuli-responsive platforms. These systems enable controlled release of H_2_S in terms of location, timing, and dosage, offering significantly improved precision and targeting.^56^ Beyond smart delivery systems, emerging strategies also encompass multi-responsive platforms, gas-prodrug-mediated delivery, and biomimetic cell-based carriers.^57^ For example, a multi-sulfonate supramolecular hydrogel triggered by UV radiation has been developed to release H_2_S on demand, demonstrating effective UV protection, good cytocompatibility, and anti-inflammatory activity.^58^ Such a system holds promising research prospects as a topical protective agent against UV radiation.

## Protective Mechanism and Application of Carbon Monoxide

### Protective effects across electromagnetic radiation injury models

#### Protection against visible light injury

The retinal protective capacity of CO is particularly salient in protecting the retina, a tissue highly vulnerable to high-energy visible light. Studies indicate that CO effectively mitigates blue light-induced damage in human retinal pigment epithelial cells by inhibiting the cleavage of key apoptotic proteins, stabilizing mitochondrial membrane potential, and activating the Nrf2 transcription factor along with its downstream antioxidant genes such as heme oxygenase-1, thereby counteracting light-triggered oxidative stress. Concurrently, CO-releasing molecules (CORMs) can exert anti-inflammatory effects by blocking NF-κB nuclear translocation.^51^,^59^ Findings from retinal cell models collectively reveal that CO acts through synergistic antioxidant, anti-inflammatory, and anti-apoptotic pathways, offering a novel therapeutic strategy for preventing intense light-induced retinopathy. This mechanistic profile is corroborated by *in vivo* evidence showing that intravitreal delivery of a CORM after retinal ischemiareperfusion injury significantly protects retinal ganglion cells. The protection is mediated through the downregulation of key apoptotic (caspase3) and proinflammatory (NFκB, and tumor necrosis factorα) mediators, coupled with the modulation of MAPK signaling pathways. Crucially, this protective profile transcends acute cytoprotection to include the enhancement of the expression of the growth-associated protein43 and promotes the regenerative potential of retinal neurons, indicating CO’s dual role in neuroprotection and repair within the central nervous system.^60^

#### Protection against ultraviolet radiation injury

CO exhibits immunomodulatory and anti-inflammatory effects in the context of UV radiation exposure. By inhibiting terminal respiratory enzymes, CO shifts cells toward a more reduced state, thereby attenuating UV-induced damage.^61^ A study demonstrated that CO alleviates acute and chronic inflammatory erythema, suppresses epidermal hyperplasia, delays the onset of early tumors, reduces tumor multiplicity, promotes the regression of established tumors, and inhibits the progression of invasive malignancies in UV-irradiated skin. These protective outcomes are associated with the down-regulation of immunosuppressive factors and the enhancement of immunostimulatory mediators, potentially alongside antioxidant mechanisms, collectively contributing to its efficacy during UV-induced photocarcinogenesis.^62^ Besides, evidence suggests that CO may exert a protective influence on bystander cells within the radiation-induced bystander effect^63^; however, the specific mechanisms involved and whether such protection extends to radio waves or UV radiation require further experimental validation.

In aggregate, across both UV radiation and high-intensity visible light-induced injury, CO interacts with multiple pathways, including oxidative stress, inflammation, cell death, and aberrant neovascularization, highlighting its potential as an emerging gaseous protective agent against electromagnetic radiation damage.

### Core molecular protective mechanisms

CO exerts its core protective functions, modulating cellular energy metabolism, suppressing excessive inflammation, and inhibiting apoptosis, by targeting the mitochondrial respiratory chain and key inflammatory signaling hubs. The cytoprotective effects of CO stem from its specific binding to heme-containing iron proteins and the subsequent activation of a multi-layered signaling network. CO binds reversibly to cytochrome c oxidase, exhibiting a concentration-dependent biphasic effect.^64^,^65^ Mild inhibition of cytochrome c oxidase at low CO concentrations induces the generation of moderate levels of mitochondrial ROS, which act as critical signaling molecules to activate adaptive programs including the Nrf2 transcription factor, thereby cooperatively maintaining mitochondrial quality and metabolic homeostasis.^66^ In regulating inflammation and immune responses, CO inhibits the initiation signaling of Toll-like receptors, downregulates NADPH oxidase activity to reduce ROS bursts, and effectively blocks NF-κB nuclear translocation and transcriptional activity. It also suppresses the assembly and activation of the NLRP3 inflammasome, ultimately leading to decreased release of pro-inflammatory cytokines.^67^,^68^ Beyond these actions, CO activates kinase pathways such as p38 MAPK and PI3K/Akt, and modulates signal transducer and activator of transcription 3, Bcl-2 family proteins, and the caspase cascade to synergistically exert anti-apoptotic and pro-survival effects.^69^ In addition to the mechanisms described above, CO engages in cross-talk with other gaseous signaling molecules such as NO and regulates cellular metabolic flux by influencing the activity of metabolic enzymes, thereby cooperatively enhancing cytoprotection.^70^ Collectively, this integrated signaling network, spanning anti-inflammatory, anti-apoptotic, and metabolic adaptive pathways, provides the molecular mechanistic foundation for its protective role against electromagnetic radiation-induced damage.

### Delivery strategies and application challenges

Initial therapeutic attempts centered on the inhalation of low-concentration CO gas, which could achieve systemic effects but posed challenges such as dose inaccuracy and risk of whole-body overexposure. Subsequent efforts shifted toward CORMs, progressing from metal-based CORMs to metal-free organic-framework-based systems. Recent advances enable visible-light-regulated, precise CO release accompanied by fluorescent signal changes, allowing for real-time visual tracking. In parallel, biomarker-responsive triggerable CORMs have been developed; these can first detect intracellular markers associated with oxidative stress and then, via a logic-gate mechanism, initiate CO release. Additionally, breakthroughs have been made in targeted delivery strategies, such as conjugating CORMs with polymeric micelles for more precise localization.^71^,^72^ At the clinical translation level, beyond conventional CORM applications, innovative systems combining three-dimensional-printed devices with CORMs have been designed. For example, a silicon-membrane-integrated module can regulate CO release rates, enabling targeted delivery without systemic exposure risks.^73^,^74^ Nevertheless, CO delivery still faces several challenges, including a lack of non-invasive methods for monitoring tissue-specific CO concentrations, variable cellular responses to CO across different tissues, and insufficient data on long-term exposure safety. Prospective research must prioritize the development of organ-specific formulations, exploring the interplay between CO and other gaseous mediators, and establishing early-prediction systems for potential CO-related complications.

## Protective Mechanism and Application of Nitric Oxide

### Pivotal role in ultraviolet radiation response and injury

NO’s biological outcome, whether protective or detrimental, is not primarily dictated by the wavelength of radiation, but by the local concentration dynamics and the resultant redox microenvironment.

#### The concentration-dependent duality

The duality of NO is most evident in UV radiation models, where it can switch from a cytoprotectant to a cytotoxic agent. Studies indicate that following UVB exposure, NO exerts a clear anti-apoptotic effect via the cyclic guanosine monophosphate (cGMP)-dependent signaling pathway.^75^,^76^,^77^ While inhibition of endogenous NO synthesis exacerbates UV-induced apoptosis, supplementation with exogenous NO donors or overexpression of inducible NO synthase significantly mitigates this damage. Experiments using endothelial and inducible NO synthase knockout mice have further validated this protective role of NO.

Conversely, radiation-triggered overstimulation of inducible NO synthase leads to a sustained overproduction of NO. Excess NO reacts rapidly with superoxide anions to form peroxynitrite, a potent oxidizing and nitrating agent. Peroxynitrite can induce lipid peroxidation and DNA damage, and through deleterious modifications promote mitochondrial dysfunction and activation of apoptotic signaling, thereby converting NO into a cytotoxic mediator.^78^,^79^

#### Mediation of immunomodulation and the bystander effect

Apart from localized cellular impacts, NO suppresses local immune function and propagates long-range bystander effects. Under UV radiation, NO contributes to local immunosuppression. This is achieved through its synergy with neuropeptides (e.g., calcitonin gene-related peptide) released from sensory nerve endings, which collectively inhibit the antigenpresenting function of epidermal Langerhans cells.^80^,^81^

More significantly, NO is established as a central signaling molecule in the radiation-induced bystander effect. Radiation stimulates irradiated cells to release NO, which diffuses to neighboring bystander cells. Through post-translational modifications such as Snitrosylation or tyrosine nitration, NO can modulate key DNA repair proteins, suppress high-fidelity homologous recombination repair, and ultimately increase genomic instability.^82^

Critically, this effect involves complex crosstalk with pathways such as HIF1α within the tumor microenvironment, where NO may differentially regulate HIF1α in irradiated versus bystander cells, exacerbating damage in the latter.^83^ The NO signaling network further interacts with other stress pathways and gaseous mediators, adding to its remarkable regulatory complexity^84^ (**Figure 4**).

**Figure 4:**
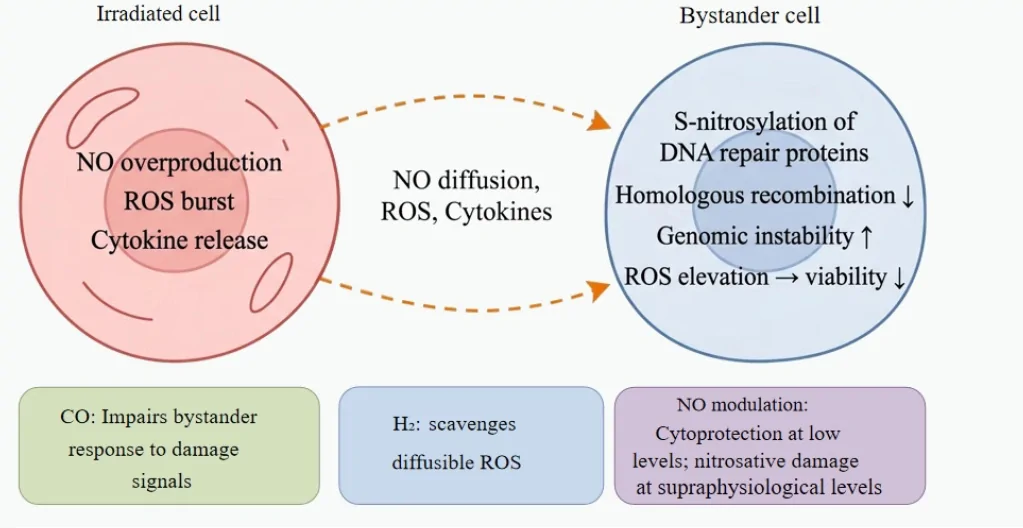
Molecular mechanisms of the radiation-induced bystander effect and potential intervention by medical gases. In the irradiated cell, NO overproduction and ROS burst trigger cytokine release and NO diffusion to adjacent bystander cells. Within bystander cells, ROS elevation reduces viability, while snitrosylation of DNA repair proteins suppresses homologous recombination, leading to genomic instability and sustained cytokine signaling. Medical gases modulate these events: CO impairs bystander response to damage; NO modulation offers cytoprotection at low levels but induces nitrosative damage at supraphysiological levels; H_2_ scavenges diffusible ROS signals, potentially mitigating bystander injury during radiotherapy. Created with Adobe Illustrator 2025. CO: carbon monoxide; H_2_: hydrogen; H_2_S: hydrogen sulfide; NO: nitric oxide; ROS: reactive oxygen species.

Collectively, these findings depict the multifaceted role of NO in the response to electromagnetic radiation. It acts as a cytoprotective agent under specific conditions, participating in anti-apoptosis and immunomodulation; yet under radiation stress, it can also become a toxic mediator and serve as a long-range messenger that disseminates damage signals to neighboring cells, amplifying the biological effects of radiation. Therefore, understanding the concentration-dependent duality of NO is crucial for further exploring its role in cellular signaling, paving the way for subsequent discussions on its core molecular mechanisms.

### Core molecular protective mechanisms

NO acts as a double-edged sword in biological systems. At low concentrations, NO activates the cGMP/PKG pathway and confers protective S-nitrosylation, thereby exerting antioxidant, anti-inflammatory, and anti-apoptotic effects; it can also act as a radical scavenger to neutralize superoxide anions.^85^ Consequently, the therapeutic modulation of NO necessitates a precise titration of its levels to leverage its S-nitrosylation-mediated protection while averting peroxynitrite-driven cytotoxicity.

### Delivery strategies and application challenges

The application of NO is historically hampered by its volatile pharmacokinetics. Although traditional NO donors can store and release NO, they generally suffer from poor stability, nontargeted release, and difficult-to-control liberation, which severely limit their clinical utility. Recent research has introduced nanotechnologybased solutions. Carrier systems represented by nanomaterials such as graphene, liposomes, and hydrogels can encapsulate NO donors to achieve targeted delivery, enhance stability, and enable ondemand controlled release, thereby significantly improving therapeutic efficacy while reducing systemic toxicity.^86^,^87^ Nevertheless, clinical translation in this field still faces critical challenges, including the need for a deeper understanding of material fate and longterm biosafety, as well as the precise spatiotemporal regulation of NO release to maximize therapeutic benefit and minimize offtarget toxicity.

## Protective Mechanism and Application of Hyperbaric Oxygen

### Protective role against ultraviolet radiation injury

HBO confers robust protection against UVA-induced dermal injury, primarily by leveraging its hormetic potential.^88^ HBO exerts its protective effects via a hormetic mechanism, mobilizing cellular antioxidant defenses to counteract UVA-mediated oxidative injury. A moderately hyperoxic environment activates the expression of Nrf2-regulated antioxidant genes, thereby enhancing cellular antioxidant capacity.^88^ Additionally, HBO upregulates NO synthase expression, which promotes vasodilation, improves tissue perfusion, accelerates the clearance of harmful metabolites, and suppresses the release of pro-inflammatory cytokines, collectively mitigating UVA-triggered inflammatory responses.^88^ HBO administered twice or four times per week significantly reduced the proportion of proliferation and apoptosis markers in the skin of UVA-exposed mice, reversed pathological alterations such as increased skin wrinkling and loss of elasticity, and downregulated both the expression and activity of caspase-3 in the liver, indicating systemic-level protection.^88^ Although the complete molecular network remains to be fully elucidated, current evidence clearly demonstrates that HBO integrates multiple pathways, including antioxidant enhancement, anti-inflammatory, and improved tissue perfusion, offering a novel therapeutic perspective for protection against UV radiation-induced damage.

### Core protective mechanisms

HBO therapy exerts its protective effects by inducing a controlled oxidative stress that activates endogenous adaptive and protective cellular networks centered on Nrf2 and HIF-1α, thereby coordinately enhancing antioxidant capacity, suppressing inflammation, and promoting tissue repair. HBO-induced ROS serve as signaling molecules that stimulate the activation of the transcription factor Nrf2, leading to the upregulation of a broad spectrum of antioxidant genes and a systematic reinforcement of cellular redox homeostasis.^89^ The intermittent hyperoxic-normoxic cycling characteristic of HBO regimens mimics the signaling of hypoxic preconditioning, which activates HIF-1α and drives the expression of genes, such as vascular endothelial growth factor, thereby promoting adaptive angiogenesis. Furthermore, HBO therapy positively modulates mitochondrial function, contributing to enhanced cellular bioenergetics. In terms of anti-inflammatory action, HBO suppresses NF-κB activation, downregulates the expression of multiple pro-inflammatory cytokines, and balances NO signaling through bidirectional regulation of NO synthase, thereby preventing nitrosative stress.^90^,^91^

### Delivery strategies and application challenges

The delivery of HBO therapy needs to establish evidence-based treatment parameters, ensure accessibility and patient compliance, and conduct large-scale trials to definitively prove efficacy for specific radiation injury indications. However, the existing literature universally reveals significant heterogeneity in treatment protocols, which presents a central challenge for clinical application and evaluation.

## Comparative Analysis and Integrated Perspectives of Different Medical Gases

The functional divergence of these five gases is fundamentally dictated by their origin, endogenous or exogenous, which in turn shapes their molecular logic and translational pathway. The endogenous gasotransmitters (CO, NO, and H_2_S) exert protection by integrating into and modulating precise cellular signaling networks. Their efficacy is not merely a function of concentration but of context-dependent signal fidelity. For instance, H_2_S and CO both converge on the Keap1-Nrf2-ARE axis to boost antioxidant defenses, but via distinct chemical dialogues.^92^ NO’s quintessential duality is mediated by the switch between cGMP-dependent signaling and nitrosative stress pathways.^93^ This inherent complexity means their therapeutic application faces the paradox of harnessing potentially toxic molecules, demanding strategies that can mimic the spatial and temporal dynamics of physiological signaling. In contrast, the exogenous agents H_2_ and HBO operate through more direct biophysical mechanisms. H_2_ acts as a selective radical scavenger and a broad-spectrum modulator of pathways like PI3K/Akt and Nrf2,^26^ but it does not engage in classical receptor-based signaling cascades. HBO induces a state of controlled oxidative stress that transcriptionally activates systemic defense programs, prominently involving the Nrf2 and HIF-1α networks.^88^,^90^,^91^ Their primary translational challenges are therefore distinct, centering on achieving sufficient deliverable doses, standardizing administration, and ensuring long-term safety for externally applied interventions.

The existing evidence base, interpreted through this mechanistic lens, shows complementary strengths. H_2_’s broad safety profile and multi-pathway modulation underpin its robust, albeit relatively non-specific, protective effects across radiofrequency and UV models.^26^ H_2_S and CO in UV-induced skin damage via deep anti-inflammatory and metabolic reprogramming,^47^,^59^ and NO as a central yet double-edged mediator in UV response and bystander effects. HBO stands apart, offering preconditioning-induced systemic resilience. A critical consensus gap for nearly all gases is the lack of data against infrared and visible light injury.

This review also reveals an underexplored intersection between gas signaling and the radiationinduced bystander effect. Gaseous mediators, particularly NO, can propagate injury signals from directly irradiated cells to neighboring nonirradiated cells, thereby amplifying tissue damage beyond the primary exposure site.^12^,^78^ A deeper mechanistic understanding of this propagation may open new avenues for confining radiation injury and strengthening normaltissue protection. Based on the bystander effect, future protective strategies should move beyond traditional external shielding. Instead, the focus should shift toward blocking intercellular signaling molecules or intervening in the microenvironment. Specifically, blockers can be developed to interrupt signals sent through gap junctions. For substances like ROS and cytokines, targeted antioxidants or neutralizing antibodies could be used to limit collateral damage.

Synergistic gas combinations present a compelling strategy to circumvent the limitations of monotherapy. Clinical medicine already utilizes several established gas mixtures. For example, Heliox is used to treat respiratory obstruction and prevent decompression sickness.^94^ A specific mixture of CO_2_ and O_2_ can improve cerebral blood flow in patients with cerebrovascular diseases.^95^ These successful clinical applications suggest that multi-modal gas interventions could also be effective in radiation protection. While the synergistic co-application of gases has proven to significantly improve therapeutic efficacy in the treatment of other diseases,^96^ this multi-modal intervention strategy holds broad research prospects in radiation prevention and treatment, urgently requiring further exploration into its synergistic mechanisms and clinical translation potential.

When evaluating the radioprotective efficacy of medical gases, a critical dimension beyond their effective wavebands is their potential tissue and organ specificity. This specificity originates from the distinct physicochemical properties of each gaseous molecule and the differential distribution of their molecular targets in vivo. H_2_, by virtue of its minimal molecular size and exceptional lipophilicity, possesses unparalleled biomembrane penetration and diffusivity.^36^ This capability renders it particularly adept at reaching compartmentalized organs protected by tight biological barriers, such as the brain and testes, providing a physicochemical rationale for the relatively robust evidence of its protection in the nervous and reproductive systems. In contrast, the biological effects of CO are fundamentally rooted in its high-affinity interaction with heme-containing iron proteins.^97^ These proteins are enriched in tissues with high energy metabolism and oxygen-sensing systems, which may form the structural basis for CO’s pronounced protective advantages observed in the cardiovascular system and related injury models. As for H_2_S and NO, their physiological actions are highly localized. Consequently, achieving targeted delivery via topical formulations likely represents the most direct translational pathway for harnessing their potent antioxidant and anti-inflammatory mechanisms against localized pathologies, such as UV-induced skin photoaging or visible light-induced retinal injury. In summary, the future design of strategies applying gaseous molecules for radioprotection should carefully consider the principle of matching gas properties–target organ microenvironment to maximize intervention efficiency (**Figure 5**).

**Figure 5:**
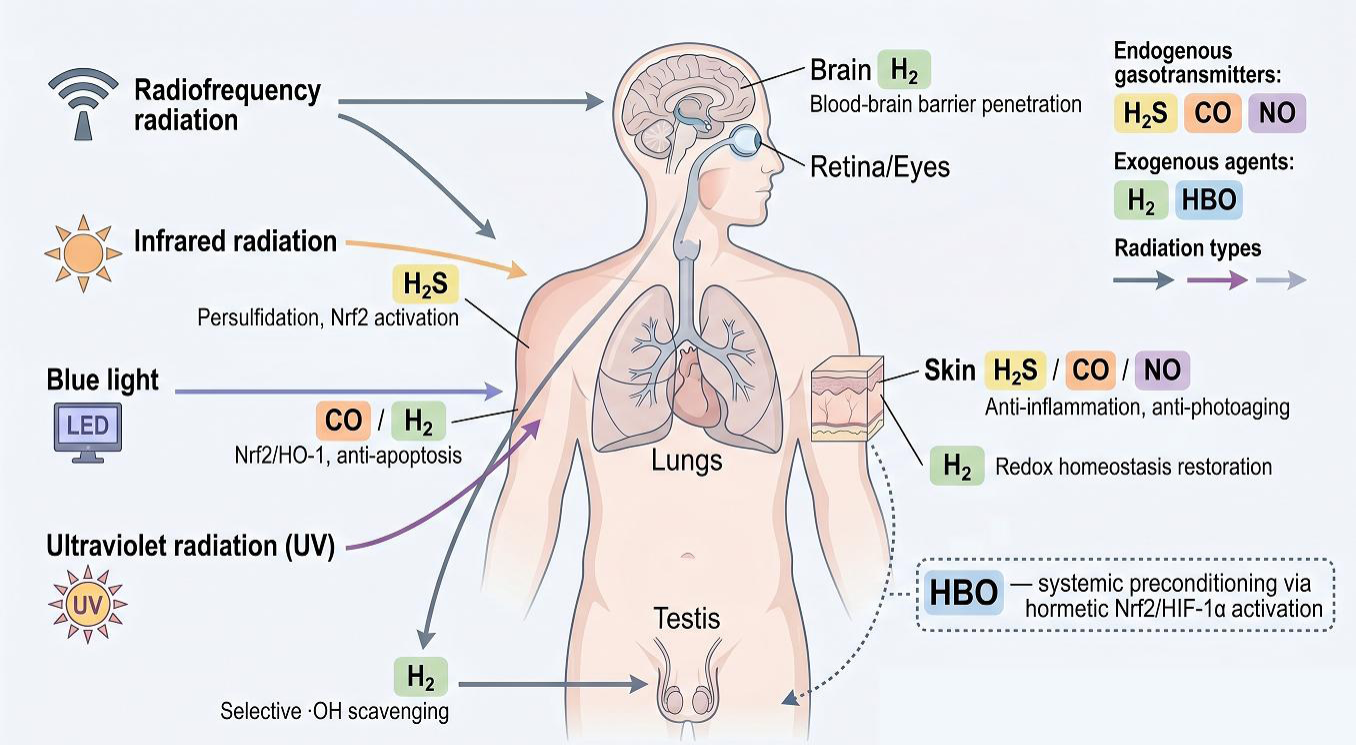
Organ-specific radioprotection by medical gases across the electromagnetic spectrum. This figure illustrates protective effects of endogenous gasotransmitters (NO, H_2_S, and CO) and exogenous agents (H_2_, and HBO) against various radiation types – from radiofrequency and infrared to blue light, LED, and UV. In the brain, H_2_ penetrates the bloodbrain barrier and provides selective ·OH scavenging. Retina/eyes benefit from H_2_S via persulfidation and Nrf2 activation. Skin protection involves H_2_S, CO, and NO, mediating antiinflammation, antiphotoaging, Nrf2/HO1, and antiapoptosis. Lungs utilize H_2_ for redox homeostasis restoration. Testis protection relies on H_2_’s selective ·OH scavenging. HBO induces systemic preconditioning through hormetic Nrf2/HIF1α activation. Together, these medical gases offer organspecific radioprotection by modulating oxidative and nitrosative stress pathways. Created with Adobe Illustrator 2025. ·OH: Hydroxyl radical; CO: carbon monoxide; H_2_: hydrogen; H_2_S: hydrogen sulfide; HBO: hyperbaric oxygen; HIF-1α: hypoxia-inducible factor 1α; HO-1: heme oxygenase-1; LED: light-emitting diode; NO: nitric oxide; Nrf2: nuclear factor erythroid 2-related factor 2; UV: ultraviolet.

## Summary and Future Perspectives

Electromagnetic radiation, as a ubiquitous environmental factor in modern society, has led to radiation-induced injury that is increasingly recognized as a public health concern. This review has systematically delineated the roles of gaseous molecules in protecting against such damage by establishing a novel, gas-centric framework (**Figure 6** and **Table 2**).

**Figure 6:**
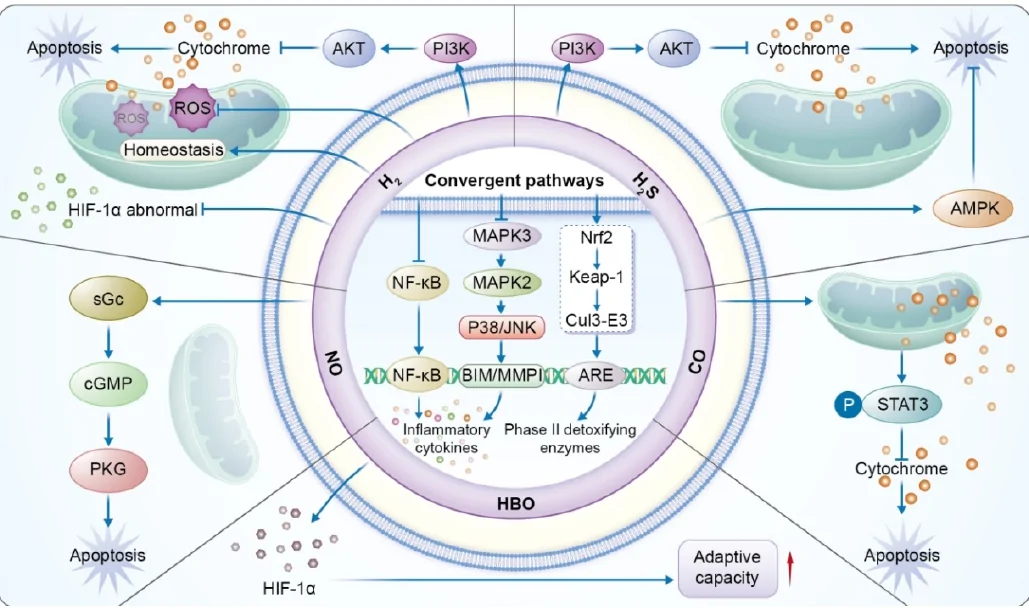
Radioprotective mechanisms of gases. Medical gases such as H_2_, H_2_S, NO, CO, and HBO exert radioprotective effects by suppressing inflammatory responses, inhibiting apoptosis, and enhancing cellular adaptive capacity via multiple signaling pathways. Created with Adobe Illustrator 2025. AKT: Protein kinase B; AMPK: adenosine monophosphate-activated protein kinase; ARE: antioxidant response element; BIM: Bcl2-interacting mediator of cell death; cGMP: cyclic guanosine monophosphate; CO: carbon monoxide; Cul3-E3: cullin 3 e3 ubiquitin ligase; H_2_: hydrogen; H_2_S: hydrogen sulfide; HBO: hyperbaric oxygen; HIF-1α: hypoxia-inducible factor 1α; JNK: c-Jun N-terminal kinase; Keap-1: Kelch-like Ech-associated protein 1; MAPK: mitogen-activated protein kinase; MMPI: matrix metalloproteinase inhibitor; NF-κB: nuclear factor κB; NO: nitric oxide; Nrf2: nuclear factor erythroid 2-related factor 2; PI3K: phosphatidylinositol 3-kinase; PKG: protein kinase G; ROS: reactive oxygen species; sGC: soluble guanylyl cyclase.

**Table 2:** Comprehensive profile of medical gases as novel radioprotective agents

Gas	Advantage	Key challenge
H_2_	1. High biocompatibility and non-toxicity	1. Suboptimal delivery efficiency
	2. Selective scavenging of ROS 3. Multi-pathway modulation 4. Relatively robust evidence for protection against radio waves and ultraviolet radiation.	2. Unclear long-term safety 3. Limited studies on efficacy against visible light and infrared radiation.
H_2_S	1. Systemic antioxidant response	1. Narrow therapeutic window; high concentrations are cytotoxic.
	2. Preliminary evidence of protection against infrared radiation and UVA	2. Conventional donors lack temporal control, posing toxicity risks. 3. Overall evidence remains sparse and fragmented across the electromagnetic spectrum
CO	1. Pronounce anti-inflammatory, anti-apoptotic, and immunomodulatory effects. 2. Document efficacy against UV-induced skin damage and high-intensity blue light-induced retinal injury.	1. Critical challenge in safety 2. Absence of non-invasive methods for real-time, tissue-specific CO monitoring
NO	1. Pivotal gaseous signaling molecule with context-dependent duality	1. Highly dependent on the radiation dose and cellular microenvironment.
	2. Low concentrations mediate protection via cGMP signal	2. Conventional NO donors suffer from instability and non-specific release.
HBO	1. Improve tissue oxygenation and perfusion, promoting repair.	1. Induce a hormetic response, systematically upregulating endogenous defenses
	2. Induce a hormetic response, systematically upregulating endogenous defenses	2. Treatment parameters require further optimization.

cGMP: Cyclic guanosine monophosphate; CO: carbon monoxide; H_2_: hydrogen; H_2_S: hydrogen sulfide; HBO: hyperbaric oxygen; NO: nitric oxide; ROS: reactive oxygen species; UV: ultraviolet.

While the therapeutic potential of medical gases is substantial, their clinical translation is impeded by a multifaceted array of pharmacological and technical constraints. A primary challenge is achieving precise spatiotemporal delivery and control. For endogenous gasotransmitters like CO and NO, their narrow therapeutic window and context-dependent duality, exemplified by NO’s shift from cytoprotection to cytotoxicity, demand delivery systems capable of mimicking physiological release within specific pathological microenvironments. Exogenous agents such as H_2_ and HBO present distinct obstacles, including maintaining effective bioactive concentrations, ensuring long-term safety, and standardizing treatment protocols. A critical, cross-cutting limitation is the lack of non-invasive methods for real-time, site-specific monitoring of gas concentrations, which is essential for dose optimization and safety. Future research and development must adopt divergent paths tailored to the origin of these agents. For endogenous gasotransmitters (CO, and NO), the priority lies in designing next-generation smart donors that respond to specific radiation-induced biomarkers to achieve spatially and temporally precise release, thereby harnessing their protective signaling while avoiding their toxic potential. In contrast, for exogenous agents (H_2_, and HBO), efforts should concentrate on optimizing delivery systems and dosing regimens to ensure reliable tissue penetration and bioavailability, alongside conducting large-scale, long-term safety studies. Ultimately, exploring synergistic combinations that leverage the rapid, direct action of exogenous agents with the sophisticated, pathway-specific modulation of endogenous gas signaling may yield the most robust radioprotective strategies.

Beyond these near-term translational efforts, more disruptive delivery concepts are beginning to emerge. One compelling idea is the use of engineered commensal bacteria that colonize the gut or skin and are programmed to release H_2_ or H_2_S on demand, potentially providing long-lasting, self-regulating protection without repeated administration.^98^ In a related vein, synthetic biology tools could equip host cells with gas-generating enzymes placed under the control of radiation-responsive promoters,^99^ thereby creating autonomous protective circuits that are activated only upon exposure. Although these approaches are still in their infancy, they illustrate how future gas-based radioprotection might expand from clinical intervention to daily prevention.

Meanwhile, broader shortcomings, including an uneven research distribution across electromagnetic wavebands, a scarcity of clinical trials, and the absence of non-invasive methods for real-time gas concentration monitoring, are comprehensively discussed. These findings must be interpreted within the context of certain methodological constraints. The scope of our synthesis was primarily restricted to English-language publications indexed in PubMed. The marked heterogeneity across studies in their experimental models, radiation parameters, and outcome endpoints made a quantitative synthesis unfeasible. Despite these limitations, the synthesis presented here offers a balanced overview of the current evidence and points to directions where more focused investigation is needed.

In conclusion, this review establishes an origin-centric framework for medical gas-based radioprotection. Moving forward, the focus must pivot toward deciphering the distinct molecular mechanisms of each gas, particularly for the less-studied candidates. Future efforts should also focus on developing tailored delivery strategies, encompassing both smart donor systems for endogenous gasotransmitters and optimized administration protocols for exogenous interventions, to ensure efficacy and safety (**Table 3**). Ultimately, exploring potential synergistic effects among multiple gases and advancing translation from basic research to clinical application will be key to transforming these promising concepts into applicable, precision biomedical strategies against electromagnetic radiation injury (**Figure 7**).

**Figure 7:**
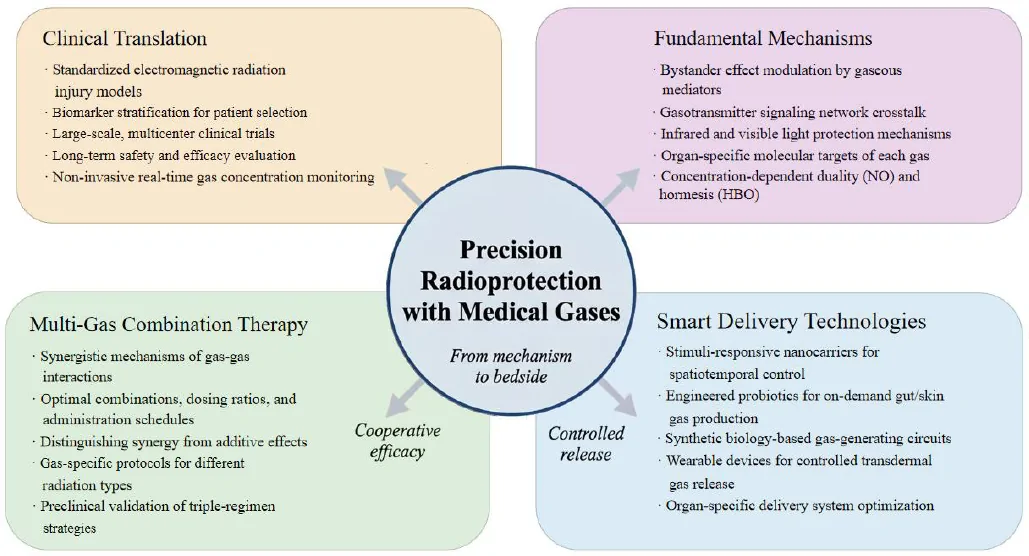
Future research agenda for medical gas-based radioprotection. This framework outlines four priority areas that will drive the next phase of research: multi-gas combination therapy, smart delivery technologies, clinical translation, and unresolved fundamental mechanisms. The central goal is to advance from mechanistic findings toward precision radioprotective strategies. Created with Adobe Illustrator 2025. HBO: Hyperbaric oxygen; NO: nitric oxide.

**Table 3:** The delivery strategies and application challenges in the protection function of gas molecules

Gas	Primary delivery method	Latest delivery strategy
H_2_	1. Inhalation	Under active development.
	2. Drinking hydrogen-rich water	
	3. Injection of hydrogen-rich saline	
	4. Topical application (H_2_-rich bath)	
H_2_S	1. Donor compounds	1. Stimuli-responsive nanosystems
	2. Prodrug design	2. Multi-response systems
	3. Stimuli-responsive nanosystems	3. Gas prodrug-mediated delivery
	4. Topical gels/dressings	4. biomimetic cell-derived carriers
		5. Polysulfide-based supramolecular hydrogels
CO	1. Low-concentration inhalation 2. Triggerable CORMs 3. Carrier-based delivery	1. Advanced formulations integrating CORMs with polymeric micelles for targeting, and 2. 3D-printed devices combined with CORMs
NO	1. NO donors	Nanocarrier-based delivery systems (e.g., liposomes)
	2. Nanocarrier delivery	
	3. Light-/enzyme-responsive release systems	
	4. Topical gaseous NO delivery	
HBO	1. Systemic HBO therapy chamber	Under development
	2. Local hyperbaric oxygen therapy	

3D: Three-dimensional; CO: carbon monoxide; CORMs: co-releasing molecules; H_2_: hydrogen; H_2_S: hydrogen sulfide; HBO: hyperbaric oxygen; NO: nitric oxide.

**Open Questions**

1. What is the ideal way to deliver each gas over time and space, and can today’s nanocarrier systems achieve this ideal delivery?
2. When exogenous gases (e.g., H_2_ and hyperbaric oxygen) and endogenous gasotransmitters (e.g., CO, NO, and H_2_S) are combined for radioprotection, do their effects work together (synergistically) or against each other (antagonistically)?
3. Why has research into medical gas radioprotection focused so much on ultraviolet and radiofrequency waves, and can the results be applied to other types of electromagnetic waves, such as infrared, microwave, and ionizing radiation?

## Data Availability

*Not available.*
